# Hydroxyethyl starch 130/0.4 for volume replacement therapy in surgical patients: a systematic review and meta-analysis of randomized controlled trials

**DOI:** 10.1186/s13741-021-00182-8

**Published:** 2021-05-11

**Authors:** Yi Xu, Siying Wang, Leilei He, Hong Yu, Hai Yu

**Affiliations:** Department of Anesthesiology, West China Hospital, Sichuan University, Chengdu, 610041 Sichuan China

**Keywords:** Hydroxyethyl starch 130/0.4, Volume replacement therapy, Surgery

## Abstract

**Background:**

The safety of perioperative intravenous hydroxyethyl starch (HES) products, specifically HES 130/0.4, continues to be the source of much debate. The aim of this meta-analysis was to update the existing evidence and gain further insight into the clinical effects of HES 130/0.4 on postoperative outcomes for volume replacement therapy in surgical patients.

**Methods:**

MEDLINE, EMBASE, and Cochrane Library databases were searched from inception to March 2020 for relevant randomized controlled trials (RCTs) on perioperative use of HES 130/0.4 in adult surgical patients. The primary outcome was postoperative mortality and secondary outcomes were the incidence of acute kidney injury (AKI) and requirement for renal replacement therapy (RRT). The analysis was performed using the random-effects method and the risk ratio (RR) with a 95% confidence interval (CI). We performed the risk-of-bias assessment of eligible studies and assessed the overall quality of evidence for each outcome.

**Results:**

Twenty-five RCTs with 4111 participants were finally included. There were no statistical differences between HES 130/0.4 and other fluids in mortality at 30 days (RR 1.28, 95% CI 0.88 to 1.86, *p* = 0.20), the incidence of AKI (RR 1.23, 95% CI 0.99 to 1.53, *p* = 0.07), or requirement for RRT (RR 0.75, 95% CI 0.37 to 1.53, *p* = 0.43). Overall, there was a moderate certainty of evidence for all the outcomes. There was no subgroup difference related to the type of surgery (*p* = 0.17) in the incidence of AKI. As for the type of comparator fluids, however, there was a trend that was not statistically significant (*p* = 0.06) towards the increased incidence of AKI in the HES 130/0.4 group (RR 1.22, 95% CI 0.97 to 1.54) compared with the crystalloid group (RR 1.21, 95% CI 0.27 to 3.91). Subgroup analyses according to the type of surgery demonstrated consistent findings.

**Conclusions:**

This systematic review and meta-analysis suggests that the use of HES 130/0.4 for volume replacement therapy compared with other fluids resulted in no significant difference in postoperative mortality or kidney dysfunction among surgical patients. Given the absent evidence of confirmed benefit and the potential trend of increased kidney injury, we cannot recommend the routine clinical use of HES 130/0.4 for volume replacement therapy in surgical patients from the perspective of benefit/risk profile. However, the results need to be interpreted with caution due to the limited sample size, and further well-powered RCTs are warranted.

**Trial registration:**

PROSPERO registry reference: CRD42020173058

**Supplementary Information:**

The online version contains supplementary material available at 10.1186/s13741-021-00182-8.

## Background

Perioperative volume replacement therapy, based on the infusion of crystalloid or colloid solutions, is ubiquitous in clinical practice and crucial to patient outcomes. Among synthetic colloids, hydroxyethyl starch (HES) solutions are by far the most studied solutions that have been used worldwide for volume replacement therapy. However, the clinical use of HES solutions has been much hampered since the reports of increased risk of kidney injury and death in critically ill patients, especially in patients with sepsis (Brunkhorst et al. 2008; Perner et al. 2012; Myburgh et al. 2012). Therefore, in 2013, both the European Medicines Agency and U.S. Food and Drug Administration recommended not to use HES solutions in critically ill patients, including those with sepsis. Nevertheless, this may not be a good and sufficient reason to ban HES from operating rooms, as surgical patients usually receive limited amounts of HES for periods of a few hours only perioperatively. Though adverse effects of HES solutions have been clearly verified in intensive care unit (ICU) patients, they have not been established in surgical patients.

In the following years, several systematic reviews and meta-analyses have indicated insufficient evidence to recommend the use of HES solutions in surgical patients (Gillies et al. 2014; Martin et al. 2013; Raiman et al. 2016; Van Der Linden et al. 2013). However, they were based on small-sampled studies and did not restrict inclusion to one particular HES product. To the best of our knowledge, the pharmacokinetic and pharmacodynamic properties of HES vary depending on molecular substitution and molecular weight, which may be associated with kidney and hemostatic function (Van Der Linden et al. 2013). Moreover, it is important to mention that modern, low-molecular-weight, low-molecular-substitution HES products, including HES 130/0.4, are commonly used nowadays and potentially associated with fewer adverse effects (Raiman et al. 2016). In addition, recently published large trials compared HES 130/0.4 with other fluids in perioperative settings, and provided conflicting results (Duncan et al. 2020; Futier et al. 2020; Kabon et al. 2019).

Therefore, we conducted a systematic review and meta-analysis to update the existing evidence and gain further insight into the effects of a modern, third-generation HES product, specifically HES 130/0.4, on postoperative mortality and renal function for volume replacement therapy in surgical patients.

## Methods

This systematic review and meta-analysis was conducted in accordance with the Preferred Reporting Items for Systematic Reviews and Meta-Analyses (PRISMA) guidelines (Moher et al. 2009). The protocol for this study is registered in the International Prospective Register of Systematic Reviews (PROSPERO) (registration number: CRD42020173058).

### Selection and exclusion criteria

We systematically searched MEDLINE, EMBASE, and Cochrane Central Register of Controlled Trials (CENTRAL) from database inception to March 1, 2020. The search strategies used are available in supplementary material Doc S1. Study inclusion criteria were as follows: (1) design: randomized controlled trials; (2) population: adult surgical patients aged 18 years or older; (3) intervention: perioperative administration of HES 130/0.4 for volume replacement therapy; (4) control: infusion of any other fluids; (5) outcomes: eligible studies must report at least one of predetermined outcomes. The primary outcome focused on postoperative mortality (within 30 days after surgery). Secondary outcomes were acute kidney injury (AKI) and requirement for renal replacement therapy (RRT) (at the longest follow-up). No language, sample size, or date of publication restrictions were applied. Exclusion criteria were as follows: (1) hemoglobin-based fluid as comparator fluids; (2) subjects undergoing organ transplantation, burns, or trauma surgery; (3) unextractable data; (4) Joachim Boldt as a named author (whose studies were retracted due to allegations of scientific misconduct) (Reilly et al. 2011). The detailed search strategy can be found in Supplementary material Doc S1. Searches were also conducted using clinical trials registry (www.clinicaltrials.gov) and Google Scholar to identify grey literature (including reports, conferences, workshop proceedings, and ongoing trials). We checked the reference list of all included studies to identify additional studies missed from the original electronic search.

### Data extraction

Two reviewers independently screened the retrieved titles and abstracts for potential inclusion, reviewed the full text of potentially eligible studies, and extracted data using a uniform data extraction form specifically developed for this review. The following data were extracted from each study: first author, title, journal, year of publication, study design, country, number of enrolled patients, distribution in both groups, inclusion criteria, comparator fluid, type of surgery, fluid therapy protocol, postoperative mortality, incidence of author-defined postoperative AKI, and requirement for RRT. Any disagreement was resolved by discussion between the two reviewers or mediated by a third reviewer.

### Risk of bias assessment

Two reviewers independently assessed risk of bias in included studies using the Cochrane Collaboration risk-of-bias tool (Higgins and Green 2011). Studies were categorized into high, low, or unclear risk of bias according to the following predefined criteria: random sequence generation (selection bias), allocation concealment (selection bias), blinding of participants and personnel (performance bias), blinding of outcome assessment (detection bias), incomplete outcome data (attrition bias), selective reporting (reporting bias), and other potential sources of bias. Each study was compared for consistency, with any disagreement resolved by discussion between the two reviewers or mediated by a third reviewer.

### Quality of evidence assessment

The overall quality of evidence for each outcome was assessed by the Grading of Recommendations, Assessment, Development and Evaluations (GRADE) system using the GRADEpro Guideline Development Tool (Software) (Guyatt et al. 2008). Meta-analysis of RCTs began as high quality of evidence and were rated down based on the following five categories: risk of bias, imprecision, inconsistency, indirectness, and publication bias. The quality of evidence was categorized as high, moderate, low, or very low. Additionally, the methodological quality of included studies was evaluated using the Jadad score, which assessed the appropriateness of randomization, blinding, and whether patient withdrawal information was provided (Jadad et al. 1996).

### Evidence synthesis and statistical analysis

As all the outcomes were dichotomous data, we presented the results as risk ratio (RR) with corresponding 95% confidence intervals (CI); to calculate the risk ratio, the total number of patients in each group and those with the event of interest were extracted from each study.

Statistical heterogeneity between studies was assessed by using *I*^2^ statistics. A *p*-value of 0.10 or less indicated considerable heterogeneity across studies. *I*^2^ values of 0 to 24.9%, 25 to 49.9%, 50 to 74.9%, and 75 to 100% suggested none, low, moderate, and high heterogeneity respectively.

The effect size of primary and secondary outcomes was analyzed with a random-effects model (DerSimonian and Laird method) to take into account clinical and methodologic diversity between studies. For outcomes with more than 10 studies, potential publication bias was assessed by visual inspection of funnel plot symmetry.

We anticipated heterogeneity across studies, therefore subgroup analysis was planned a priori according to the type of surgery (cardiac versus non-cardiac/mixed surgery). All statistical analyses and meta-analyses were performed using Review Manager (RevMan, V.5.3). A two-sided *p-*value of less than 0.05 was considered statistically significant.

## Results

### Study selection and characteristics

**Fig. 1:**
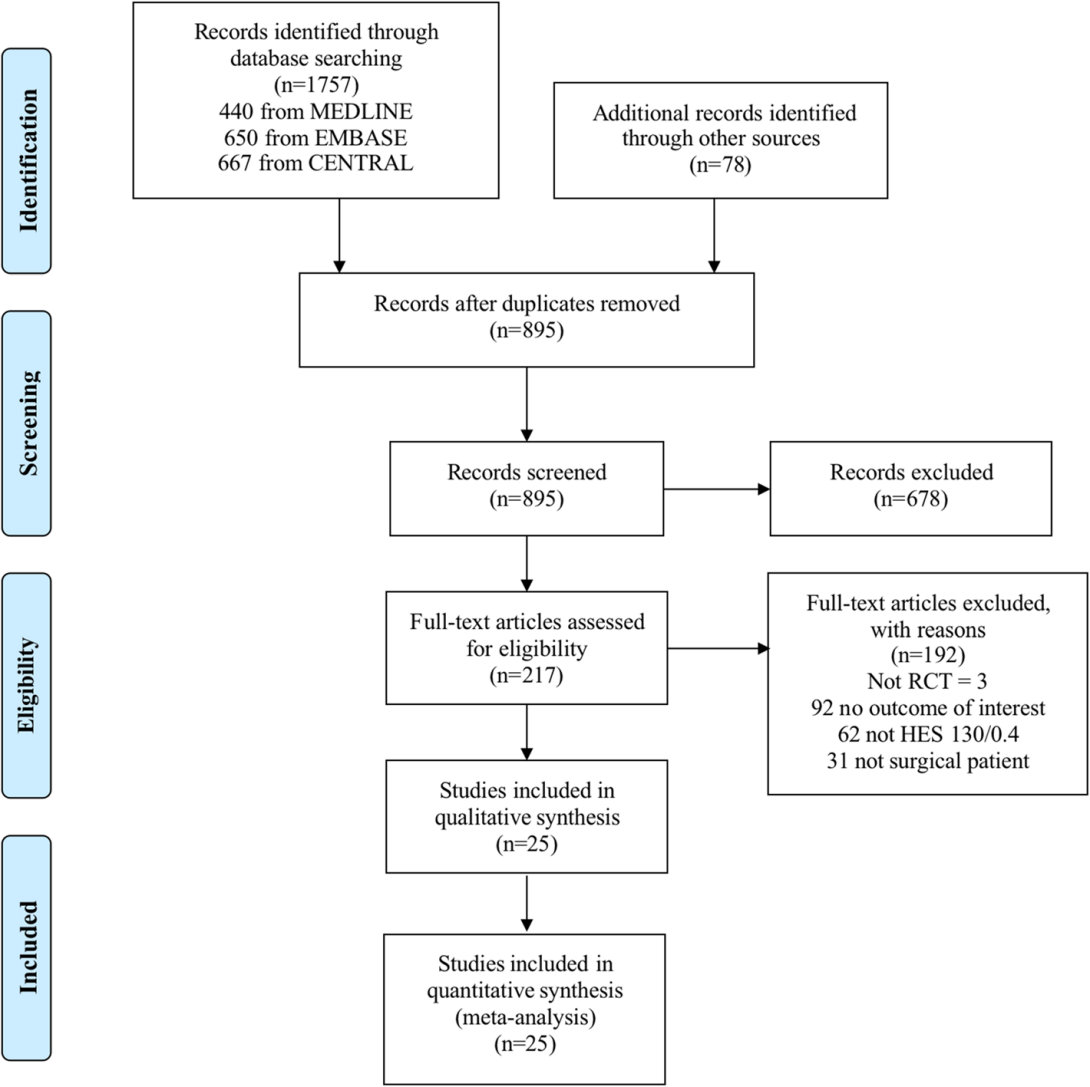
PRISMA flow diagram of trial selection

The initial electronic search retrieved 1757 citations, and the grey literature search identified additional 78 studies. This process identified 217 potentially eligible studies for full-text review. After duplicate and ineligible studies were removed, 25 RCTs with a total of 4111 participants were finally included in our systematic review and meta-analysis (Fig. 1). All included studies were published between 2005 and 2020 with a sample size from 30 to 1057 patients. Five trials were multicenter RCTs (Futier et al. 2020; Godet et al., 2008; Gondos et al. 2010; Joosten et al. 2018; Kabon et al. 2019) and the remainder were single-center RCTs. All trials but three had a Jadad score of three or more (Lee et al. 2011; Ooi et al. 2009; Yang et al. 2011). Among all the included studies, seven trials focused on patients undergoing cardiac surgery, and the study conducted by Gondos et al was in a mixed group of patients undergoing both cardiac and non-cardiac surgeries (Gondos et al. 2010). A variety of comparator fluids were used, including crystalloid solutions, HES 200/0.62, gelatin, and albumin. Additionally, the average dose of HES 130/0.4 administration ranged from 10 to 42 ml/kg. Eleven of the 25 studies used goal-directed fluid therapy (GDFT) for perioperative volume replacement. The main characteristics of included studies are summarized in Table 1, and an additional table is available in Table S1.

**Table 1:** Main characteristics of included studies

Trail	Single/multicenter	Country	Type of surgery	No. of patients	Comparators	Volume of HES 130/0.4	GDFT	Outcomes	Jadad score
Alavi, 2012	Single	Iran	Cardiac	92	RL, 4% gelatin	1320 ± 250 mL	No	Mortality	3
Duncan, 2020	Single	USA	Cardiac	141	5% HA	500 (400–750) mL	No	AKI, RRT	5
Feldheiser, 2013	Single	Germany	Gynecological	50	Balanced crystalloid	Not available	Yes	Mortality, AKI	4
Futier, 2020	Multi	France	Abdominal	775	0.9% saline	1000 (750–1500) mL	Yes	Mortality, AKI, RRT	5
Ghodraty, 2017	Single	USA	Abdominal	91	RL	10.4 ± 4.1 mL/kg	No	AKI	5
Godet, 2008	Multi	France	Vascular	65	3% gelatin	1431 ± 731 mL (19.7 ± 9.9 mL/kg)	No	Mortality, AKI, RRT	4
Gondos, 2010	Multi	Hungary	mixed	200	RL, 4% gelatin, 5% HA	10 ml/kg	No	Mortality	3
Hamaji, 2013	Single	Brazil	Orthopedic	48	RL	15 ml/kg	No	Mortality, AKI	4
Hung, 2014	Single	China	abdominal	80	RL	999.1 ± 369.3 mL	No	Mortality, AKI	4
Joosten, 2018	Multi	Belgium	Abdominal	160	Balanced crystolloid	900 (400–1300) mL	Yes	Mortality, AKI ,RRT	5
Kabon, 2019	Multi	Austria, USA	Abdominal	1057	RL	1000 (500–1500) mL	Yes	Mortality, AKI, RRT	5
Kammerer, 2018	Single	Germany	Urological	100	5% HA	2955 ± 1043 mL	No	AKI	3
Lee, 2011	Single	Republic of Korea	Cardiac	106	Crystalloid (plasma solution A)	1458 ± 465 mL	No	AKI, RRT	1
Lindroos, 2013	Single	Finland	Neurological	30	RL	464 ± 284 mL	Yes	Mortality, AKI	3
Mahmood, 2007	Single	UK	Vascular	62	HES 200/0.62, 4% gelatin	3911 ± 1783 mL	No	Mortality, RRT	3
Ooi, 2009	Single	Malaysia	Cardiac	90	4% gelatin	1942.3 ± 1046.1 mL	No	Mortality	2
Rasmussen, 2014	Single	Denmark	Abdominal	33	RL	2605 ± 512 mL	Yes	Mortality	5
Skhirtladze, 2014	Single	Austria	Cardiovascular	236	RL, 5% HA	42 (35–48) mL/kg 2500 (2250 –2750) mL	No	RRT	5
Szturz, 2014	Single	Czech Republic	Urological	115	RL	1500 (400–3500) mL	Yes	Mortality	5
Tyagi, 2019	Single	India	Orthopedic	38	RL	689 ± 394 mL	Yes	AKI	5
Van der Linden, 2005	Single	Belgium	Cardiac	132	3% gelatin	21.3 ± 8.3 mL/kg	Yes	Mortality	3
Verheij, 2006	Single	Netherlands	Cardiovascular	67	0.9% saline, 4% gelatin, 5% HA	1600 (750–1800) mL	No	Mortality	3
Yang, 2011	Single	China	Abdominal	90	RL, 20% HA	3484.6 ± 1072.5 mL	No	Mortality	2
Yates, 2014	Single	UK	Abdominal	202	Balanced crystalloid	1875 (1500–3000) mL	Yes	Mortality, AKI	5
Zhang, 2012	Single	China	Abdominal	60	RL	865.0 ± 297.4 mL	Yes	Mortality, AKI	5

*RL* Ringer’s Lactate solution, *HA* human serum albumin, *AKI* acute kidney injury, *RRT* renal replacement therapy, *CVP* central venous pressure, *SV* stroke volume, *SVV* stroke volume variation, *GDFT* goal-directed fluid therapy, *MAP* mean arterial pressure, *POD* postoperative day

**Fig. 2:**
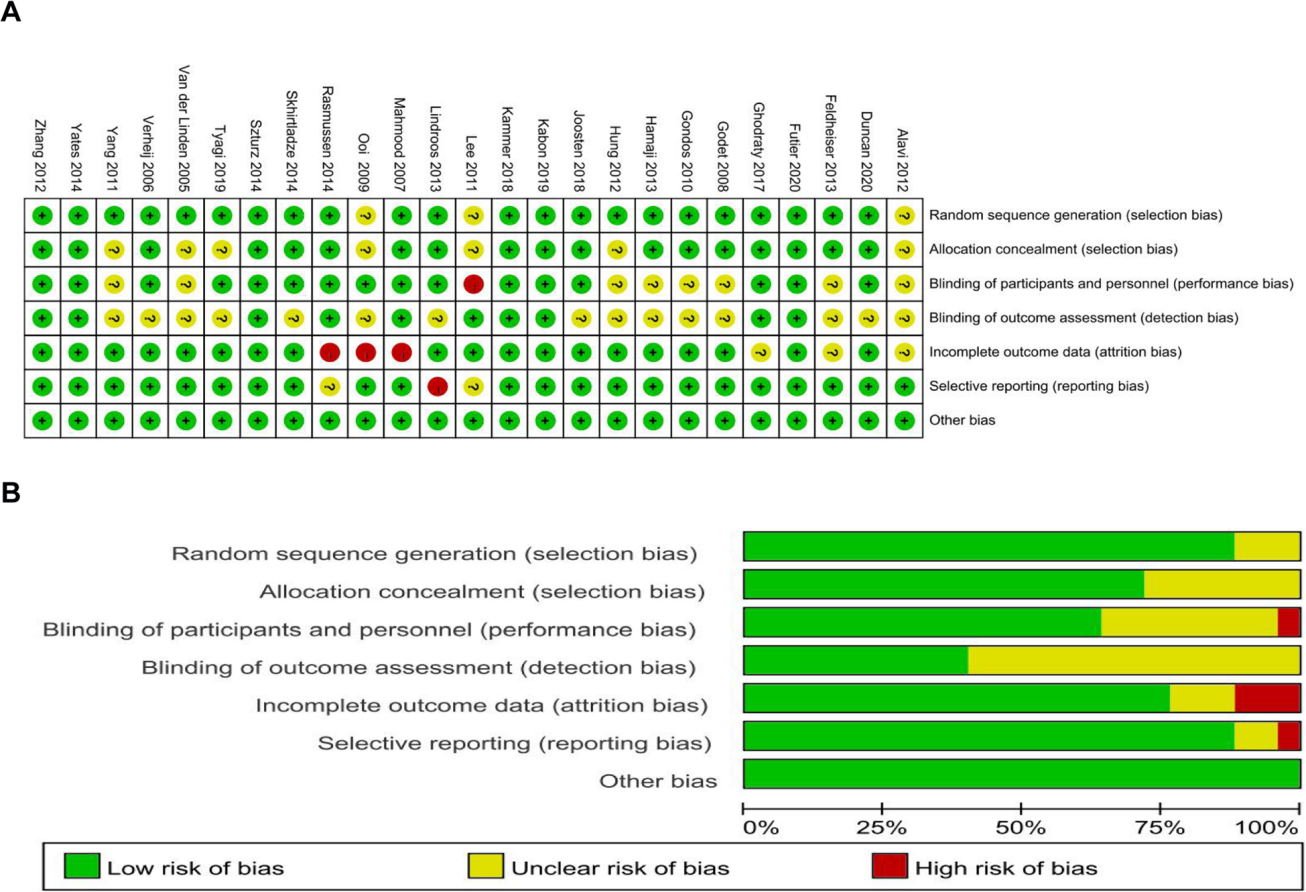
Risk of bias assessment. **a** Risk of bias summary. **b** Risk of bias graph. The plus sign indicates low risk, the minus sign high risk, and the question mark uncertain risk

Random sequence generation was present in 22 studies (88%), allocation concealment in 18 studies (72%), blinding of participants and personnel in 16 studies (64%), and blinding of outcome assessment in 10 studies (40%). Incomplete outcome data was adequately explained in 19 studies (76%), and three studies (12%) had selective reporting of outcomes. According to the Cochrane Collaboration’s risk of bias tool, six trials were judged to be low risk of bias in all domains (Futier et al. 2020; Kabon et al. 2019; Kammerer et al. 2018; Szturz et al. 2014; Yates et al. 2014; Zhang et al. 2012). Fourteen trials had unclear risk of bias, mostly related to blinding of participants and personnel and blinding of outcome assessment (Alavi et al. 2012; Duncan et al. 2020; Feldheiser et al. 2013; Ghodraty et al. 2017; Godet et al., 2008; Gondos et al. 2010; Hamaji et al. 2013; Hung et al. 2014; Joosten et al. 2018; Skhirtladze et al. 2014; Tyagi et al. 2019; Van der Linden et al. 2005; Verheij et al. 2006; Yang et al. 2011), while five trials had high risk of bias (Lee et al. 2011; Lindroos et al. 2013; Mahmood et al. 2007; Ooi et al. 2009; Rasmussen et al. 2014). The domain judged to have the highest risk of bias was attrition bias. The GRADE assessment demonstrated an overall moderate level of evidence for each outcome. The risk of bias graph and summary for the individual studies are reported in Fig. 2 and a table of individual study bias is available in the Supplementary material (Table S2).

### Primary outcome—postoperative mortality

**Fig. 3:**
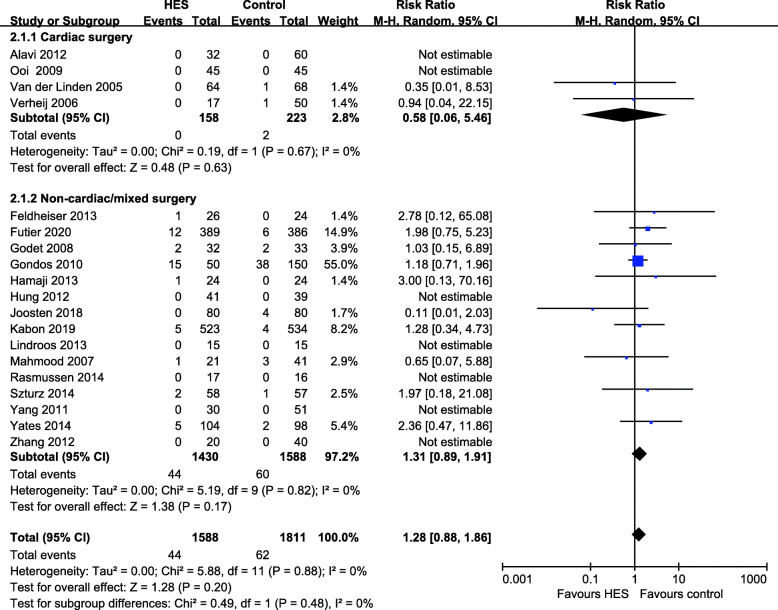
Forest plot for the effects of HES 130/0.4 versus other fluids on postoperative mortality. Subgroup analysis shows cardiac surgery versus non-cardiac/mixed surgery. HES hydroxyethyl starch, CI confidence interval, M-H Mantel–Haenszel

Nineteen studies reported mortality within 30 days after surgery (Alavi et al. 2012; Feldheiser et al. 2013; Futier et al. 2020; Kabon et al. 2019; Godet et al., 2008; Gondos et al. 2010; Hamaji et al. 2013; Hung et al. 2014; Joosten et al. 2018; Lindroos et al. 2013; Mahmood et al. 2007; Ooi et al. 2009; Rasmussen et al. 2014; Tyagi et al. 2019; Szturz et al. 2014; Van der Linden et al. 2005; Verheij et al. 2006; Yang et al. 2011; Yates et al. 2014; Zhang et al. 2012). The longest follow-up period for mortality in each study was described in supplementary material (Table S1). The overall postoperative mortality at 30 days was 44/1588 (2.8%) in the HES 130/0.4 group and 62/1811 (3.4%) in the control group, which showed no statistically significant difference between compared arms (RR 1.28, 95% CI 0.88 to 1.86, *p* = 0.20, *I*^2^ = 0%). There were no deaths in 7 of the 19 studies (Alavi et al. 2012; Hung et al. 2014; Lindroos et al. 2013; Ooi et al. 2009; Rasmussen et al. 2014; Yang et al. 2011; Zhang et al. 2012). There was no subgroup effect related to the type of surgery (*p* = 0.48). No difference was observed in either cardiac surgery group (RR 0.58, 95% CI 0.06 to 5.46, *p* = 0.63, *I*^2^ = 0%) or non-cardiac/mixed surgery group (RR 1.31, 95% CI 0.89 to 1.91, *p* = 0.17, *I*^2^ = 0%) (Fig. 3). There was no subgroup effect related to the type of comparator fluids (*p* = 0.78) (Fig. S1). Funnel plot analysis suggested visually no significant asymmetry, suggesting a low chance of publication bias (Fig. S2).

### Secondary outcomes—incidence of author-defined AKI

**Fig. 4:**
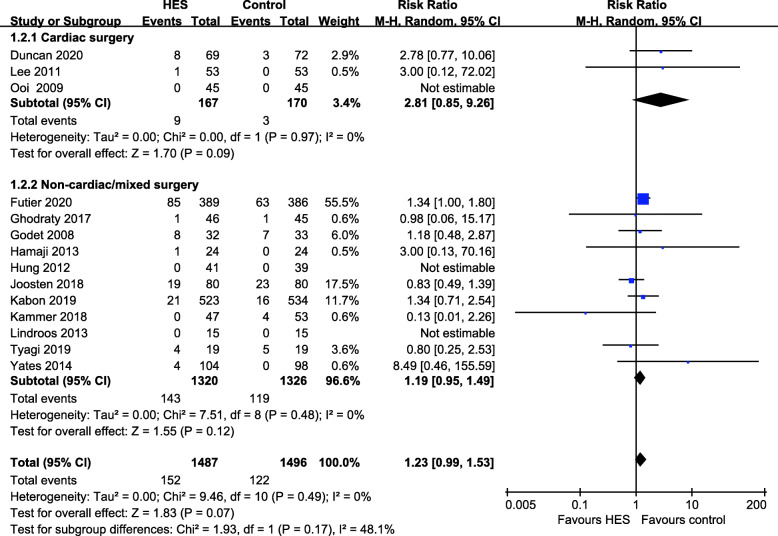
Forest plot for the effects of HES 130/0.4 versus other fluids on incidence of author-defined acute kidney injury (AKI). Subgroup analysis shows cardiac surgery versus non-cardiac/mixed surgery. HES hydroxyethyl starch, CI confidence interval, M-H Mantel–Haenszel

Author-defined AKI was reported in 15 RCTs with 3179 patients (Duncan et al. 2020; Feldheiser et al. 2013; Futier et al. 2020; Ghodraty et al. 2017; Godet et al. 2008; Hamaji et al. 2013; Hung et al. 2014; Joosten et al. 2018; Kabon et al. 2019; Kammerer et al. 2018; Lee et al. 2011; Lindroos et al. 2013; Tyagi et al. 2019; Yates et al. 2014; Zhang et al. 2012). Postoperative AKI was defined in nine studies using clinical or laboratory biomarkers including serum creatinine, glomerular filtration rates, and requirement for dialysis. Three studies used the risk/injury/failure/loss/end-stage (RIFLE) classification (Hamaji et al. 2013; Kammerer et al. 2018; Bellomo et al. 2004), and one study was based on the definition by the Acute Kidney Injury Network (AKIN) grade (Ghodraty et al. 2017; Mehta et al. 2007). Three studies used the Kidney Disease Improving Global Outcomes (KDIGO) criteria (Futier et al. 2020; Joosten et al. 2018; Tyagi et al. 2019; Kellum et al. 2013). All three of these consensus definitions define AKI based either on the increase in serum creatinine or on the change of urine output. The incidence of author-defined AKI in the HES 130/0.4 group was 10.0% (152/1513) and 7.9% (122/1551) in the comparison group (RR 1.23, 95% CI 0.99 to 1.53, *p* = 0.07, *I*^2^ = 0%), a difference that was not statistically significant but that displayed a potential trend towards better renal protection by the comparator fluid. In four studies, no patient developed AKI (Hung et al. 2014; Lindroos et al. 2013; Ooi et al. 2009; Yang et al. 2011). As for the type of surgery, there was not statistically significant difference (*p* = 0.17) between the cardiac surgery group (RR 2.81, 95% CI 0.85 to 9.26, *p* = 0.09, *I*^2^ = 0%) and non-cardiac/mixed surgery group (RR 1.19. 95% CI 0.95 to 1.49, *p* = 0.12, *I*^2^ = 0%) (Fig. 4). In addition, there was no subgroup difference related to the type of comparator fluids (*p* = 0.99) (Fig. S3).

### Secondary outcomes—incidence of requirement for RRT

**Fig. 5:**
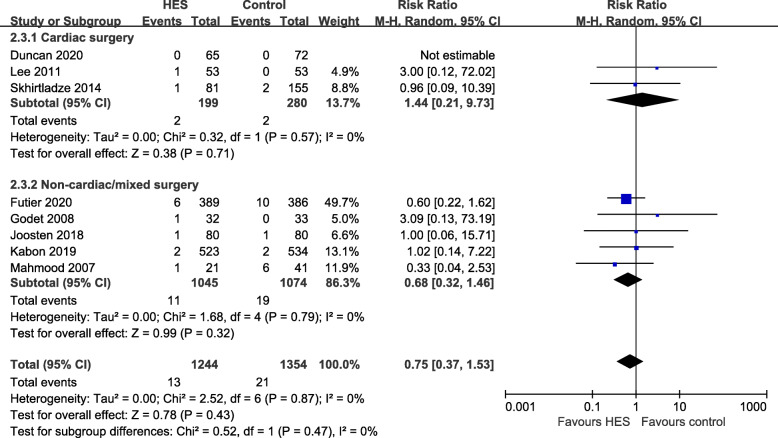
Forest plot for the effects of HES 130/0.4 versus other fluids on incidence of requirement for renal replacement therapy (RRT). Subgroup analysis shows cardiac surgery versus non-cardiac/mixed surgery. HES hydroxyethyl starch, CI confidence interval, M-H Mantel–Haenszel

Based on data from eight RCTs including 2597 participants (Duncan et al. 2020; Futier et al. 2020; Godet et al., 2008; Joosten et al. 2018; Kabon et al. 2019; Lee 2011; Mahmood et al. 2007; Skhirtladze et al. 2014), there was no statistically significant difference between the two groups in requirement for RRT (RR 0.75, 95% CI 0.37 to 1.53, *p* = 0.43), and no heterogeneity (*p* = 0.87, *I*^2^ = 0%). The number of patients with requirement for RRT was 13/1244 (1.0%) in the HES 130/0.4 group and 21/1351 (1.6%) in the comparison group. There was no need for RRT in one study (Duncan et al. 2020). There was no subgroup effect in terms of the type of surgery (*p* = 0.47). With regard to subgroup analysis, cardiac surgery (RR 1.44, 95% CI 0.21 to 9.73, *p* = 0.71, *I*^2^ = 0%) and non-cardiac/mixed surgery (RR 0.68, 95% CI 0.32 to 1.46, *p* = 0.32, *I*^2^ = 0%) did not result in a statistically significant difference (Fig. 5). There was no subgroup effect related to type of comparator fluids (*p* = 0.64) (Fig. S4)

## Discussion

This systematic review and meta-analysis showed no significant difference in postoperative mortality or renal dysfunction (the incidence of AKI and requirement for RRT) between HES 130/0.4 and other fluids in surgical patients for volume replacement therapy. These findings were consistent in subgroup analyses of patients undergoing cardiac or non-cardiac surgery.

Intravenous fluid therapy is of paramount importance in perioperative care. Given the potential benefits of HES, including longer intravascular persistence than crystalloids and relatively lower price than albumin, it has been used widely for intravascular volume maintenance or augmentation for decades (Finfer S. 2013). However, the use of HES has been suspended since several large trials reported its detrimental clinical effects in critically ill and septic patients, with a potential increased risk of renal dysfunction and death (Brunkhorst et al. 2008; Perner et al. 2012; Myburgh et al. 2012; Zarychanski et al. 2013). Unlike critically ill patients, however, surgical patients present an intact tight glycocalyx/vascular endothelial junction for the retention of colloids, whereas endotoxic shock or sepsis in critically ill patients impairs the vascular endothelium integrity resulting in substantial extravasation of large molecules (Steppan et al. 2011). Also, surgical patients typically receive limited amounts of HES over just a few hours only perioperatively. Until now, no definite conclusions have been drawn regarding the safety of HES for volume replacement therapy during the perioperative period.

We found no statistically significant difference in postoperative mortality. This result is in line with meta-analyses by Raiman in 2016 and Gillies in 2014. It is important to highlight that the duration of follow-up and postoperative morality have certain relevance. Compared with long-term mortality, short-term mortality is a rare event, and the statistical power of this outcome could be lower. An earlier RCT demonstrated that the hospital mortality and 3-month mortality in the HES group were 0% and 19% respectively (Feldheiser et al. 2013). On the contrary, a recent large multicenter RCT did not demonstrate a significant difference in mortality at days 14, 28, or 90 when comparing HES with 0.9% saline (Futier et al. 2020). Previous meta-analyses by Gillies in 2014 and Raiman in 2016 evaluated the hospital mortality and 90-day mortality respectively. Owing to the relatively short duration of follow-up in most of the included studies, our systematic review focuses on short-term mortality (within 30 days after surgery). Of all the RCTs we identified, only the trial by Duncan et al evaluated the effect of HES on long-term outcomes (i.e. 1-year mortality) (Duncan et al. 2020). Future studies should consider evaluating the safety of HES 130/0.4 on long-term mortality in a surgical setting. It is possible that we identify a negligible difference in short-term mortality but a substantial difference in long-term mortality attributable to higher event rates and corresponding greater statistical power.

To the best of our knowledge, concerns about the use of HES have been raised mainly about an increased risk of kidney injury, which consequently could lead to increased mortality. Older generations of HES characterized by higher molecular weight induce tubular swelling and osmotic necrosis due to cytoplasmic vacuole formation thereby causing renal toxicity (Nan et al. 2005). On the contrary, the newer generation of HES with lower molecular weight is thought to have an improved safety profile (Westphal et al. 2009). Our meta-analysis considered the existence of differences between the different generations of HES and focused on the third-generation product, specifically HES 130/0.4. Importantly, whereas no statistical difference was identified regarding incidence of AKI, there may be a potential trend towards increased AKI by HES 130/0.4 when compared with the comparator fluid (RR 1.23, 95% CI 0.99 to 1.53, *p* = 0.07). It is notable that the study by Kammerer et al is the outlier, so we tested the effect on the meta-analysis calculation after the results of this study were excluded. Interestingly, there was nominally higher incidence of AKI in HES 130/0.4 group compared with the comparator, although the difference was not statistically significant (RR 1.24, 95% CI 1.00 to 1.55, *p* = 0.05) (Fig. S5). In addition, we chose clinical outcome measures such as AKI and RRT instead of biochemical markers, such as serum creatinine values, calculated creatinine clearance, urinary neutrophil gelatinase-associated lipocalin and cystatin C ratio, to assess the renal safety. Nevertheless, data describing renal adverse events were not well reported. Only 7 of 25 included studies reported AKI using diverse internationally defined criteria including RIFLE, AKIN and KIDGO Classifications (Futier et al. 2020; Duncan et al. 2020; Ghodraty et al.^2017^; Hamaji et al. 2013; Joosten et al. 2018; Kammerer et al. 2018; Tyagi et al. 2019) (Table S2), which is a potential source of heterogeneity in this meta-analysis. Moreover, the US Food and Drug Administration has suggested that kidney function monitored for at least 90 days in all patients receiving HES solutions. Therefore, the short observation period for AKI in this study may have missed some adverse kidney events induced by HES (Xue et al. 2020). Therefore, standardized optimal definition staging for AKI should be reported and be systematically followed up to facilitate the comparison and combination of trials.

Predefined subgroup analyses were performed on patients undergoing cardiac surgery, because cardiopulmonary bypass (CPB) induced inflammation, endothelial dysfunction and abnormal microvascular permeability may augment the risk of kidney dysfunction and mortality (Dabbagh et al. 2012). However, the systemic inflammatory response from CPB and sepsis seemed to differ (Butler et al. 1993). Tassani et al. demonstrated that the CPB-induced inflammatory response did not result in an alteration of protein distribution, and capillary leak syndrome associated with CPB was not observed (Tassani et al. 2002). Consequently, the safety of HES may differ when used in the cardiac surgery setting from critically ill patients or non-cardiac surgery. To address possible confounders, we also featured subgroup analyses according to type of comparator fluids (i.e. crystalloids and non-crystalloids). In the current meta-analysis, the primary and secondary outcomes did not differ significantly between subgroups.

It is noteworthy that the study by Futier in 2020, i.e. FLASH trial, is an important contribution to this topic. The findings from their study demonstrated no advantages of using HES 130/0.4 for volume replacement therapy in high-risk patients undergoing abdominal surgery. Furthermore, the FLASH trial even suggested concerns about the detrimental clinical effects of HES 130/0.4 for patients in several settings (both the ICU and the operating room). As the lack of demonstrable benefits, the authors did not support the use of HES in the perioperative period. Newly-published propensity score-matched cohort studies suggested that HES 130/0.4 administration during surgery was not significantly associated with postoperative renal dysfunction or mortality (Miyao et al. 2020; Lee et al. 2020). Given the inconclusive current evidence, it is worth waiting for the results of large multicenter RCTs in the future, such as the HOENICS trial, which will provide clinically relevant information regarding the safety and efficacy of HES 130/0.4 and probably have a major impact on its future.

Although the type of fluid plays certainly a role, the volume of fluid infused and the timing of administration are of importance. In the majority of included studies, the maximum daily dose of HES was 50 ml/kg or less, which limited the potential harm to patients from high doses of HES. Furthermore, over the decades, GDFT has remained a high area of interest in perioperative medicine. GDFT allowed strict standardization of fluid administration under recommended and validated protocols, thereby improving patient outcomes. Is the use of HES in the defined limits safe? Does this fluid provide any clinical benefit in comparison to other fluids in the context of GDFT in surgical patients? It is difficult to determine until further investigations. Several high-quality studies have standardized the volume and timing of administration using a GDFT protocol. (Futier et al. 2020; Joosten et al. 2018; Kabon et al. 2019). Joosten et al even developed a closed-loop fluid administration system provided by a minimally-invasive hemodynamic monitoring device and controlled by a computer. However, the use of HES solution according to a GDFT algorithm compared with other fluids resulted in no significant difference in postoperative mortality and complications.

The strengths of this review include a comprehensive search strategy using major biomedical databases for published data and grey literature, and a focus on clinically relevant outcomes. Secondly, we followed a rigorous methodology. The review of eligibility criteria, data extraction, and outcome assessment were all performed in duplicate with a high degree of inter-rater agreement. Additionally, the risk-of-bias for each trial and quality of evidence for each outcome were evaluated. To address potential confounders, we also featured prespecified subgroup analyses and sensitivity analyses. Thirdly, this review contained the largest number of RCTs published on this topic, which allowed outcomes to meet the optimal information size and allowed us to make more reliable inferences. Fourthly, no significant statistic heterogeneity was detected according to the *I*^2^ statistics. Finally, publication bias was assessed visually using a funnel plot (Fig. S1), and no obvious asymmetry was detected for the primary outcome.

Several potential limitations are also present in this meta-analysis. First, the majority of included studies were small single-center RCTs, with low event rates for both death and AKI, which resulted in insufficient statistical power to detect the difference in outcomes. In fact, although there is no evidence of statistical heterogeneity regarding mortality and morbidity, these results have to be interpreted with caution given the small sample size of many studies. Second, several important variables may be the sources of clinical heterogeneity between studies, including definition of AKI, type of surgical procedure, type of comparator fluids, and fluid therapy strategy. However, to address these possible confounders, subgroup analysis was performed a priori to adjust the confounding factor of cardiac surgery and non-cardiac surgery. Apart from this, we subdivide our analysis regarding the type of comparator fluids, i.e. crystalloids versus non-crystalloids, thereby mitigating the risk of creating a spurious finding. Lastly, all outcomes were rated down for imprecision as the 95% CI included appreciable benefit or harm. Overall, there was a moderate certainty of evidence for each outcome.

## Conclusions

This systematic review and meta-analysis comprehensively evaluated the safety of HES 130/0.4 for volume replacement therapy in surgical patients. After including 4111 patients, we found no evidence that the perioperative administration of HES 130/0.4 for volume replacement therapy in surgical patients is associated with increased postoperative mortality, AKI or requirement for RRT. However, our findings cannot be regarded as evidence of safety. Given the insufficient and inconclusive evidence, we cannot recommend the use of HES 130/0.4 for volume replacement therapy in surgical patients. Future well-powered RCTs should focus on the effect of HES 130/0.4 on both short- and long-term outcomes in surgical patients through a hemodynamic-based, goal-directed approach, and take the volume and timing of administration into consideration.

## Supplementary Information

**Additional file 1: Doc S1.** Search strategy (DOCX)

**Additional file 2: Table S1.** Mortality at longest follow-up and definitions of acute kidney injury (AKI)

**Additional file 3:** **Table S2.** GRADE certainty

**Additional file 4:** **Figure S1.** Forest plot for subgroup analysis on postoperative mortality: Type of comparator fluids.

**Additional file 5:** **Figure S2.** Funnel plot of postoperative mortality.

**Additional file 6:** **Figure S3.** Forest plot for subgroup analysis on incidence of author-defined acute kidney injury (AKI): Type of comparator fluids.

**Additional file 7:** **Figure S4.** Forest plot for subgroup analysis on requirement for RRT: Type of comparator fluids.

**Additional file 8:** **Figure S5.** Forest plot on the incidence of author-defined acute kidney injury (AKI): Exclusion of the study by Kammerer (2018).

## Data Availability

All data generated or analyzed during this study are included in this published article.

## Abbreviations

RCT: Randomized controlled trial
AKI: Acute kidney injury
RRT: Renal replacement therapy
RR: Relative risk
CI: Confidence interval
HES: Hydroxyethyl starch
ICU: Intensive care unit
PRISMA: Preferred Reporting Items for Systematic Reviews and Meta Analyses
RIFLE: Risk/injury/failure/loss/end-stage
AKIN: Acute Kidney Injury Network
KDIGO: Kidney Disease Improving Global Outcomes
CPB: Cardiopulmonary bypass
GDFT: Goal-directed fluid therapy
