# Supplementary material for: Hydroxyethyl starch 130/0.4 for volume replacement therapy in surgical patients: a systematic review and meta-analysis of randomized controlled trials
Source: Perioper Med (Lond). 2021 May 11;10:16. doi: 10.1186/s13741-021-00182-8 (PMC8111748; doi:10.1186/s13741-021-00182-8)
Supplement: Supplementary file 1 — Additional file 1: Doc S1. Search strategy (DOCX) [file 13741_2021_182_MOESM1_ESM.docx]

**Search strategy**

**MEDLINE**：

1 Hydroxyethyl Starch Derivatives[MeSH Terms]

2 hes 130/0.4[Title/Abstract]

3 hydroxyethyl starch[Title/Abstract]

4 hydroxyethyl starch 130/0.4[Title/Abstract]

5 hydroxyethylstarch[Title/Abstract]

6 (starch, hydroxyethyl) [Title/Abstract]

7 volulyte[Title/Abstract]

8 voluven[Title/Abstract]

9 1 or 2 or 3 or 4 or 5 or 6 or 7 or 8

10 general surgery[MeSH Terms]

11 surgery[Title/Abstract]

12 surgical[Title/Abstract]

13 operation[Title/Abstract]

14 10 or 11 or 12 or 13

15 Randomized controlled trial[Publication Type]

16 Controlled clinical trial[Publication Type]

17 Randomized[Title/Abstract]

18 Randomly[Title/Abstract]

19 15 or 16 or 17 or 18

20 9 and 14 and 19

**EMBASE:**

#1 'hydroxyethyl starch derivatives'/exp OR 'hes 130/0.4':ti,ab,kw OR 'hydroxyethyl starch':ti,ab,kw OR 'hydroxyethyl starch 130/0.4':ti,ab,kw OR hydroxyethylstarch:ti,ab,kw OR 'starch, hydroxyethyl':ti,ab,kw OR volulyte:ti,ab,kw OR voluven:ti,ab,kw

#2 'surgery'/exp OR surgery:ti,ab,kw OR ‘general surgery’:ti,ab,kw OR surgical:ti,ab,kw OR operation:ti,ab,kw

#3 'randomized controlled trial'/exp OR 'controlled clinical trial':it OR 'randomized controlled trial':it OR randomized:ti,ab,kw OR randomly:ti,ab,kw

#4 #1 AND #2 AND #3

**CENTRAL:**

#1 MeSH descriptor: [Hydroxyethyl Starch Derivatives] explode all trees

#2 (hes 130/0.4):ti,ab,kw OR (hydroxyethyl starch):ti,ab,kw OR (hydroxyethyl starch 130/0.4):ti,ab,kw OR (hydroxyethylstarch):ti,ab,kw OR (starch, hydroxyethyl):ti,ab,kw OR (volulyte):ti,ab,kw OR (voluven):ti,ab,kw

(Word variations have been searched)

#3 #1 or #2

#4 (randomized controlled trial):pt OR (Controlled clinical trial):pt OR randomized:ti,ab,kw OR randomly:ti,ab,kw

(Word variations have been searched)

#5 (surgery):ti,ab,kw OR (general surgery):ti,ab,kw OR (operation):ti,ab,kw OR (surgical):ti,ab,kw

(Word variations have been searched)

#6 #3 and #4 and #5
