# Supplementary material for: Hydroxyethyl starch 130/0.4 for volume replacement therapy in surgical patients: a systematic review and meta-analysis of randomized controlled trials
Source: Perioper Med (Lond). 2021 May 11;10:16. doi: 10.1186/s13741-021-00182-8 (PMC8111748; doi:10.1186/s13741-021-00182-8)
Supplement: Supplementary file 2 — Additional file 2: Table S1. Mortality at longest follow-up and definitions of acute kidney injury (AKI) [file 13741_2021_182_MOESM2_ESM.docx]

**Table S1 Mortality at longest follow-up and definitions of AKI**

| Trial | Mortality time points | Definitions of AKI |
| --- | --- | --- |
| Alavi, 2012 | In-hospital | No |
| Duncan, 2020 | 1 year | RIFLE criteria |
| Feldheiser, 2013 | In-hospital | No |
| Futier, 2020 | 14 day | KDIGO criteria |
| Ghodraty, 2017 | No | AKIN grade I or greater based on increase in SCr ≥0.3 mg/dL |
| Godet, 2008 | In-hospital | SCr above the upper limit of normal plus an increase of ≥44.2μmmol/L (≥0.5 mg/dL) above baseline |
| Gondos, 2010 | In ICU | No |
| Hamaji, 2013 | In-hospital | RIFLE criteria |
| Hung, 2012 | In-hospital | RRT in hospital |
| Joosten, 2018 | 30 day | KDIGO or the requirement of RRT |
| Kabon, 2019 | 30 day | Requiring dialysis |
| Kammer, 2018 | 90 day | RIFLE criteria |
| Lee, 2011 | No | one or more of the following: increase in SCr to >2.0mg/dl; ≥50% decrease in the eGFR compared to the baseline; and a new requirement for dialysis |
| Lindroos, 2013 | In hospital | Not stated |
| Mahmood, 2007 | In hospital | No |
| Ooi, 2009 | 4 week | SCr >106 mmol/kg (males) and >480 mmol/kg (females) |
| Rasmussen, 2014 | In-hospital | SCr > 300 µmol/L in hospital |
| Skhirtladze, 2014 | 90 day | No |
| Szturz et al^34^ (2014) | In-hospital | Oliguria (<500ml/day), SCr increase >30% from preoperative creatinine, or urinary catheter for nonsurgical reason in hospital |
| Trial | Mortality time points | Definitions of AKI |
| Szturz, 2014 | No | KDIGO criteria |
| Tyagi, 2019 | In-hospital | No |
| Van der Linden, 2005 | In-hospital | No |
| Verheij, 2006 | In-hospital | Renal failure in hospital |
| Yang, 2011 | In-hospital | Requiring continuous veno-venous hemofiltration (CVVH) or dialysis |
| Yates, 2014 | In-hospital | No |

*Abbreviations: AKI* acute kidney injury, *RRT* renal replacement therapy, *SCr* serum creatinine, *eGFR* estimated glomerular filtration rates; *AKIN* Acute Kidney Injury Network; *RIFLE* Renal risk, Injury, Failure, Loss of kidney function, End-stage kidney disease; *KDIGO* Kidney Disease: Improving Global Outcomes
