# Supplementary material for: Hydroxyethyl starch 130/0.4 for volume replacement therapy in surgical patients: a systematic review and meta-analysis of randomized controlled trials
Source: Perioper Med (Lond). 2021 May 11;10:16. doi: 10.1186/s13741-021-00182-8 (PMC8111748; doi:10.1186/s13741-021-00182-8)
Supplement: Supplementary file 3 — Additional file 3: Table S2. GRADE certainty [file 13741_2021_182_MOESM3_ESM.docx]

**Table S2** GRADE certainty

**(A)** Summary of findings for outcomes

| **Hydroxyethyl starch 130/0.4 for volume replacement therapy in surgical patients** | | | | | |
| --- | --- | --- | --- | --- | --- |
| **Patient or population:** surgical patients **Settings:** perioperative setting **Intervention:** Hydroxyethyl starch 130/0.4 | | | | | |
| **Outcomes** | **Illustrative comparative risks* (95% CI)** | | **Relative effect (95% CI)** | **No of Participants (studies)** | **Quality of the evidence (GRADE)** |
|  | Assumed risk | Corresponding risk |  |  |  |
|  | **Control** | **Hydroxyethyl starch 130/0.4** |  |  |  |
| **postoperative mortality** Follow-up: 0-30 days | **Study population** | | **RR 1.28**  (0.88 to 1.86) | 3399 (19 RCTs) | ⨁⨁⨁◯ **moderate**^1^ |
|  | **34 per 1000** | **44 per 1000** (30 to 64) |  |  |  |
| **incidence of author-defined AKI** Follow-up: 0-1 year | **Study population** | | **RR 1.23**  (0.99 to 1.53) | 3064 (15 RCTs) | ⨁⨁⨁◯ **moderate**^2^ |
|  | **79 per 1000** | **97 per 1000** (78 to 120) |  |  |  |
| **requirement for RRT** Follow-up: 0-1 year | **Study population** | | **RR 0.75**  (0.37 to 1.53) | 2597 (8 RCTs) | ⨁⨁⨁◯ **moderate**^3^ |
|  | **16 per 1000** | **12 per 1000** (6 to 24) |  |  |  |
| *The **corresponding risk** (and its 95% CI) is based on the assumed risk in the comparison group and the **relative effect** of the intervention (and its 95% CI). **CI:** Confidence interval; **RR:** Risk ratio; **AKI:** acute kidney injury; **RRT:** renal replacement therapy | | | | | |
| **GRADE Working Group grades of evidence** **High quality:** Further research is very unlikely to change our confidence in the estimate of effect.  **Moderate quality:** Further research is likely to have an important impact on our confidence in the estimate of effect and may change the estimate. **Low quality:** Further research is very likely to have an important impact on our confidence in the estimate of effect and is likely to change the estimate. **Very low quality:** We are very uncertain about the estimate. | | | | | |
| Explanations ^1^ The 95% CI includes no effect and a potential 86% relative risk increase. ^2^ The 95% CI shows both no effect and relative risk increase of 53%. ^3^ The 95% CI includes is too wide, crossing both threshold of benefit and harm. | | | | | |

**(B)** Evidence profile for outcomes

| **Quality assessment** | | | | | | | **No of patients** | | **Effect** | | **Quality** | **Importance** |
| --- | --- | --- | --- | --- | --- | --- | --- | --- | --- | --- | --- | --- |
|  |  |  |  |  |  |  |  |  |  |  |  |  |
| **No of studies** | **Design** | **Risk of bias** | **Inconsistency** | **Indirectness** | **Imprecision** | **Other considerations** | **Hydroxyethyl starch 130/0.4** | **Control** | **Relative (95% CI)** | **Absolute** |  |  |
| **postoperative mortality (follow-up 0-30 days)** | | | | | | | | | | | | |
| 19 | RCTs | no serious | no serious | no serious | serious^1^ | none | 44/1588  (2.8%) | 62/1811  (3.4%) | **RR 1.28**  (0.88 to 1.86) | 10 more per 1000  (from 4 fewer to 29 more) | **⨁⨁⨁◯** **MODERATE** | CRITICAL |
| **incidence of author-defined AKI (follow-up 0-1 year)** | | | | | | | | | | | | |
| 15 | RCTs | no serious | no serious | no serious | serious^2^ | none | 152/1513  (10%) | 122/1551  (7.9%) | **RR 1.23**  (0.99 to 1.53) | 18 more per 1000  (from 1 fewer to 42 more) | **⨁⨁⨁◯** **MODERATE** | CRITICAL |
| **requirement for RRT (follow-up 0-1 year)** | | | | | | | | | | | | |
| 8 | RCTs | no serious | no serious | no serious | serious^3^ | none | 13/1244  (1%) | 21/1353  (1.6%) | **RR 0.75**  (0.37 to 1.53) | 4 fewer per 1000  (from 10 fewer to 8 more) | **⨁⨁⨁◯**  **MODERATE** | IMPORTANT |

^1^ The 95% CI includes no effect and a potential 86% relative risk increase.
^2^ The 95% CI shows both no effect and relative risk increase of 53%.
^3^ The 95% CI includes is too wide, crossing both threshold of benefit and harm.
