# Supplementary figures and images for: Hydroxyethyl starch 130/0.4 for volume replacement therapy in surgical patients: a systematic review and meta-analysis of randomized controlled trials
Source: Perioper Med (Lond). 2021 May 11;10:16. doi: 10.1186/s13741-021-00182-8 (PMC8111748; doi:10.1186/s13741-021-00182-8)

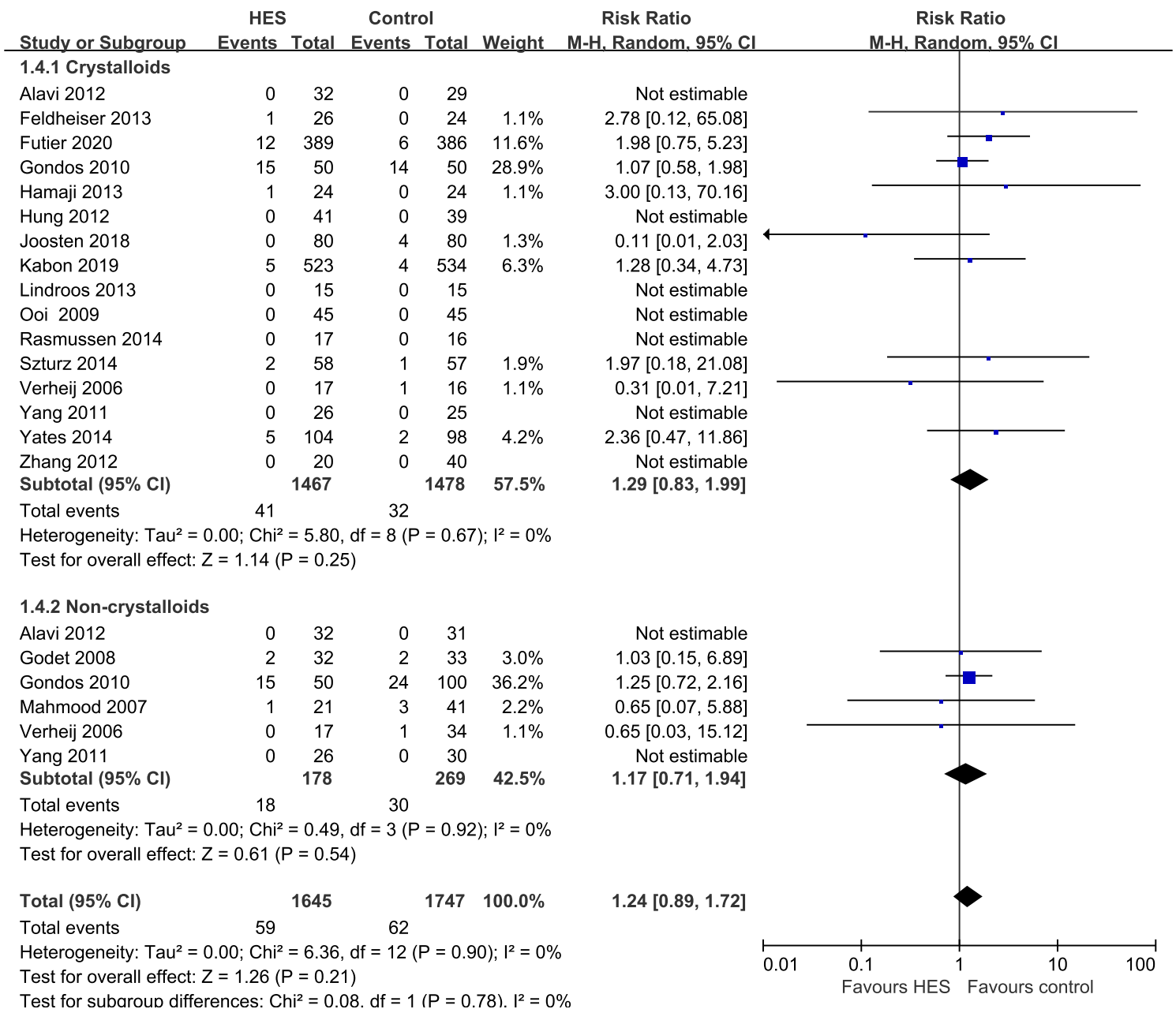

Figure S1. Subgroup analysis on postoperative mortality: Type of comparator fluids

Supplement: Supplementary file 4 — Additional file 4: Figure S1. Forest plot for subgroup analysis on postoperative mortality: Type of comparator fluids. [file 13741_2021_182_MOESM4_ESM.pdf]

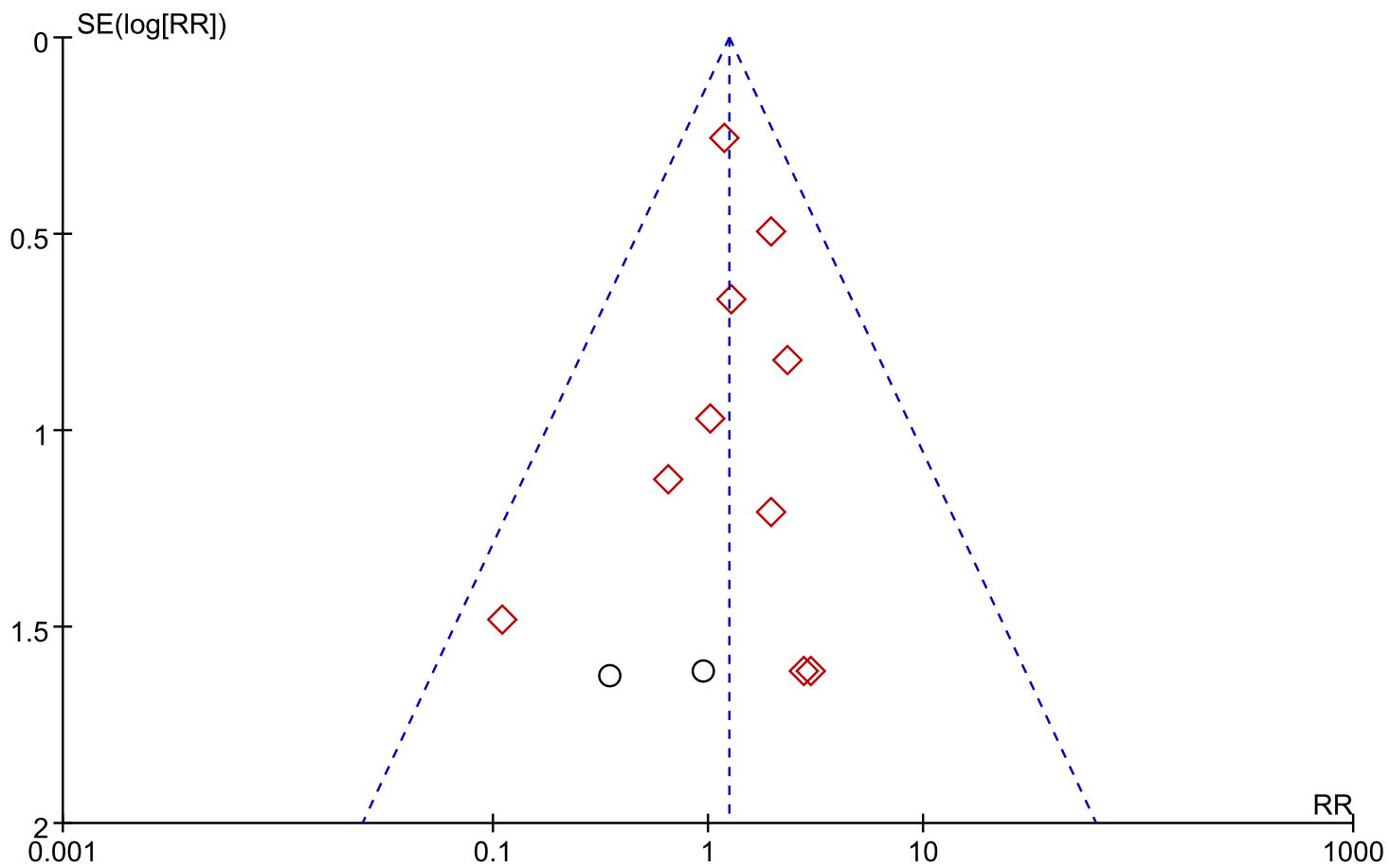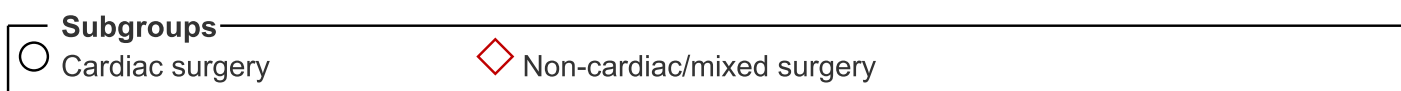

Supplement: Supplementary file 5 — Additional file 5: Figure S2. Funnel plot of postoperative mortality. [file 13741_2021_182_MOESM5_ESM.pdf]

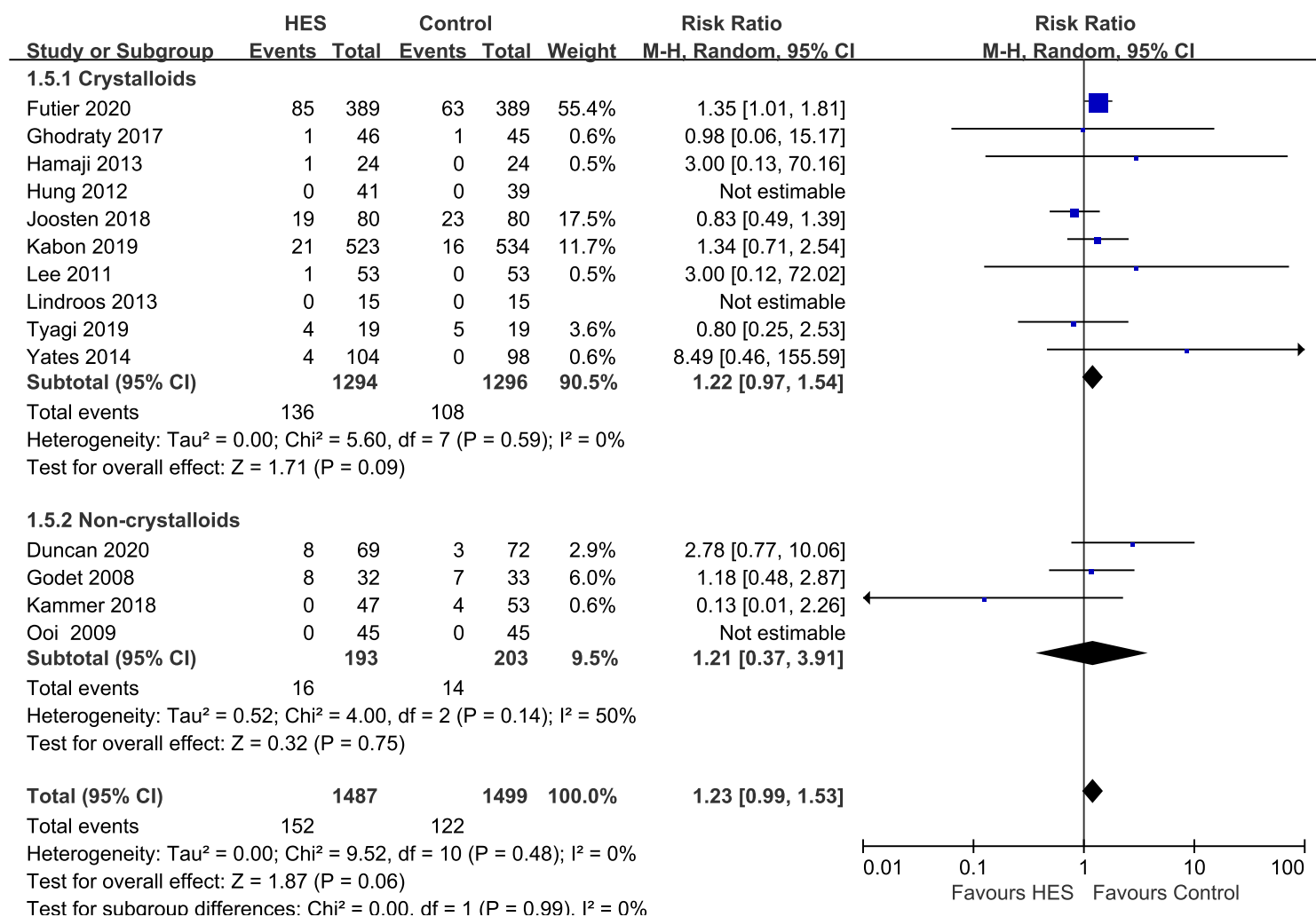

Fig. S3. Subgroup analysis on incidence of author-defined AKI: Type of comparator fluids

Supplement: Supplementary file 6 — Additional file 6: Figure S3. Forest plot for subgroup analysis on incidence of author-defined acute kidney injury (AKI): Type of comparator fluids. [file 13741_2021_182_MOESM6_ESM.pdf]

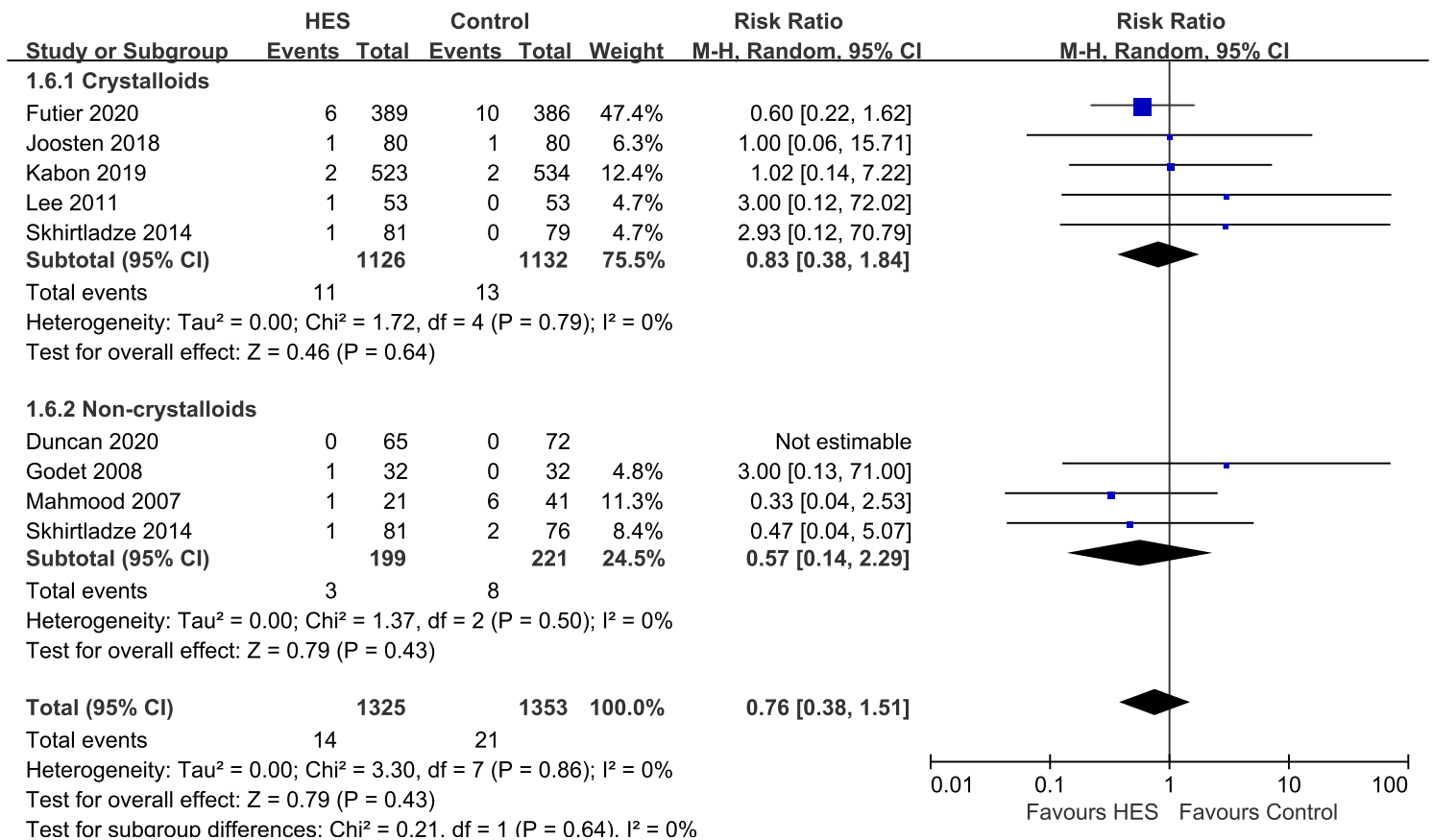

Fig. S4. Subgroup analysis on requirement for RRT: Type of comparator fluids

Supplement: Supplementary file 7 — Additional file 7: Figure S4. Forest plot for subgroup analysis on requirement for RRT: Type of comparator fluids. [file 13741_2021_182_MOESM7_ESM.pdf]

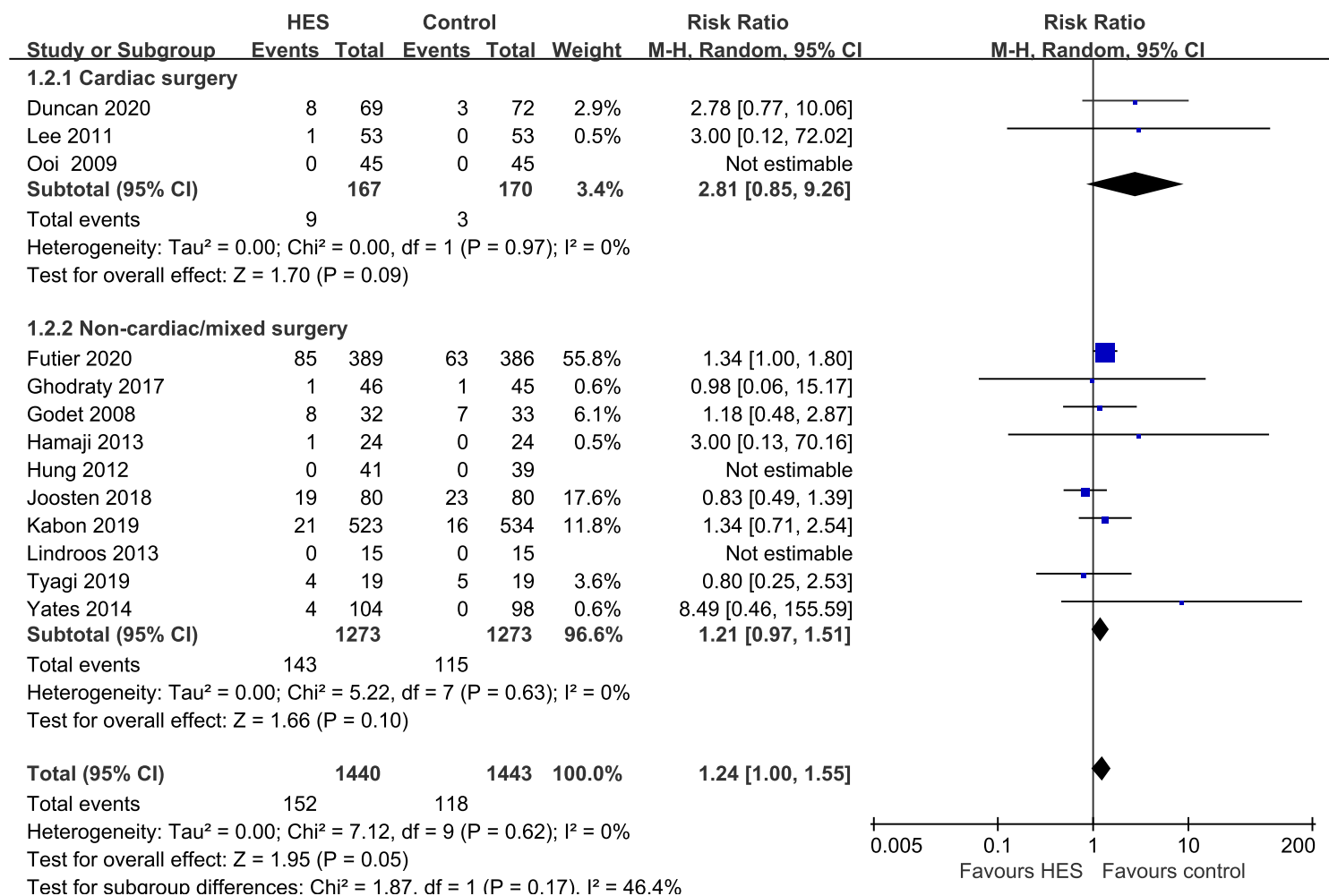

Fig. S5. Forest plot on the incidence of AKI: Exclusion of the study by Kammerer (2018)

Supplement: Supplementary file 8 — Additional file 8: Figure S5. Forest plot on the incidence of author-defined acute kidney injury (AKI): Exclusion of the study by Kammerer (2018). [file 13741_2021_182_MOESM8_ESM.pdf]
